# Antiproliferative Effect of Ascorbic Acid Is Associated with the Inhibition of Genes Necessary to Cell Cycle Progression

**DOI:** 10.1371/journal.pone.0004409

**Published:** 2009-02-06

**Authors:** Sophie Belin, Ferdinand Kaya, Ghislaine Duisit, Sarah Giacometti, Joseph Ciccolini, Michel Fontés

**Affiliations:** 1 EA 4263, Therapy of Genetic Disorder, Faculté de Médecine de la Timone, Marseille, France; 2 UPRES EA 3286, Laboratory of Pharmacokinetic and Toxicokinetic, Faculté de Pharmacie, Marseille, France; Istituto Nazionale Tumori, Italy

## Abstract

**Background:**

Ascorbic acid (AA), or Vitamin C, is most well known as a nutritional supplement with antioxidant properties. Recently, we demonstrated that high concentrations of AA act on *PMP22* gene expression and partially correct the Charcot-Marie-Tooth disease phenotype in a mouse model. This is due to the capacity of AA, but not other antioxidants, to down-modulate cAMP intracellular concentration by a competitive inhibition of the adenylate cyclase enzymatic activity. Because of the critical role of cAMP in intracellular signalling, we decided to explore the possibility that ascorbic acid could modulate the expression of other genes.

**Methods and Findings:**

Using human pangenomic microarrays, we found that AA inhibited the expression of two categories of genes necessary for cell cycle progression, tRNA synthetases and translation initiation factor subunits. In *in vitro* assays, we demonstrated that AA induced the S-phase arrest of proliferative normal and tumor cells. Highest concentrations of AA leaded to necrotic cell death. However, quiescent cells were not susceptible to AA toxicity, suggesting the blockage of protein synthesis was mainly detrimental in metabolically-active cells. Using animal models, we found that high concentrations of AA inhibited tumor progression in nude mice grafted with HT29 cells (derived from human colon carcinoma). Consistently, expression of tRNA synthetases and ieF2 appeared to be specifically decreased in tumors upon AA treatment.

**Conclusions:**

AA has an antiproliferative activity, at elevated concentration that could be obtained using IV injection. This activity has been observed *in vitro* as well *in vivo* and likely results from the inhibition of expression of genes involved in protein synthesis. Implications for a clinical use in anticancer therapies will be discussed.

## Introduction

For a long time, ascorbic acid (AA) has been described as a molecule absolutely required for the integrity and normal life span in mammalians. Most of princeps studies have been derived from the observation on patients suffering from scurvy. Numerous articles have been published demonstrating the necessity of supplement human nutrition with AA, either originating from foods or from nutrition complements. Different biochemical properties have been attributed to AA, essentially in relation with its well-documented antioxidant activity. However, little is known about the effects of AA treatment on gene expression.

In previous studies we showed that high concentrations of AA down-regulate the cAMP-dependent expression of *PMP22*, partially correcting Charcot-Marie Tooth disease phenotype in mouse models [1]. In additional experiments, we demonstrated this action relies on the AA-induced reduction of intracellular pool of cAMP [2]. Importantly, other antioxidants like retinol and α-tocopherol were unable to modulate the pool [3], strongly suggesting the inhibitory effect of AA was unrelated to its antioxidant properties. Indeed, we have recently reported that AA is a competitive inhibitor of adenylate cyclase, probably due to partial three-dimensional structures similitude between AA and ATP [4].

Because of the critical role of cAMP in the modulation of gene expression, we sought to determine whether other genes could be sensitive to AA treatment. We conducted a series of *in vitro* and *in vivo* experiments using varying concentrations of AA. We first analyzed the impact on gene expression using human pangenomic microarrays. As we found that several genes implicated in cell proliferation were underexpressed in presence of AA, we further analyzed the effect of AA administration on cell division and tumor progression. Data presented here strongly indicate that AA has an antiproliferative activity, potentially due to the inhibition of expression of genes involved in cell division progression and may be related to its action as a “global regulator” of intracellular cAMP pool.

## Materials and Methods

### Cells and culture conditions

The human skin fibroblast cell line was obtained from the Coriell Cell Repository. All others cells were purchased from the American type Culture Collection. Cells were grown according to the manufacturer’s instructions in RPMI 1640 with 25 mM HEPES supplemented with 15% fetal bovine serum (GIBCO) at 37°C in 5% CO2/95% air. Cells were counted on a microscope (10× objective) using a Neubauer hemocytometer. Cell viability was estimated using trypan blue staining.

### AA Treatment

L(+) ascorbic acid (99.7%, Riedel-de-Haën) was dissolved as needed in 1× PBS and then filtered. It was added to a final concentration of 50 mg/ml in pH 7 medium supplemented with 25 mM HEPES.

### RNA extraction and analysis

Following the incubation period, total RNA was extracted from cells using TRIZOL reagent (Invitrogen) in accordance with the manufacturer’s instructions. To ensure the integrity of the RNA prior to use, the quality and concentration of each sample was tested using an Agilent 2100 Bioanalyzer.

### Hybridization and microarray scanning

The following materials were purchased from Agilent Technologies: Low RNA Input Fluorescent Linear Amplification Kit, in situ hybridization kit, and 60-mer human oligomicroarray kit. The reverse transcription labelling reactions and hybridizations essentially followed the protocol recommended by Agilent Technologies version 4.1 (2004).

Briefly, a 500-ng aliquot of each total RNA sample was reverse transcribed into cDNA using an oligo(dT) primer.

The reaction was carried out in a solution containing 500 μM dNTP and 400 U MMLV reverse transcriptase at 40°C for 2 h, and then terminated by incubation at 65°C for 15 min. Reverse transcription and incorporation were performed at 40°C for 2 h in a mixture containing either 400 μM cyanine 5-carbamylated protein (Cy3 for the untreated samples) or 400 μM cyanine 5-carbamylated protein (Cy5 for the treated samples), the transcription mix, and T7 RNA polymerase. RNeasy spin columns (Qiagen) were used according to the manufacturer’s protocol to purify the amplified labelled cDNA samples. Hybridization was carried out in 440 μl of mixture containing 750 ng of Cy3- and Cy5-labeled cDNA probes, control targets, and the fragmented DNA supplied by the manufacturer at 60°C for 17 h. The microarrays were then washed according to the manufacturer’s protocol, dried, and scanned using an Agilent microarray scanner with 532 nm laser for Cy3 measurement and a 635 nm laser for Cy5 measurement. The results were analyzed using the Luminator Bioinformatics platform (Rosetta Biosoftware). Results have been deposited in MIAMExpress data base (accession number E-MEXP-1861).

### Reverse transcriptase-PCR analysis

To confirm the gene expression patterns observed on the microarrays we verified gene expression levels following AA treatment at each concentration by quantitative reverse transcriptase-PCR using the Light Cycler 480 Real-Time PCR System (Roche) with universal probe library (UPL). Two micrograms of total RNA from each sample were reverse transcribed using Superscript II reverse transcriptase (Invitrogen) to produce cDNA. Human β-actin (*ACTB*) and 18S human ribosomal DNA were used for data normalization. The results were treated using the comparative C_T_ method, where the amount of the target, normalized to the endogenous reference and relative to a calibrator, is given by 2^−ΔΔCT^ [5]. (An asterisk indicates a *P* value<0,05).

### Cell cycle and death analysis—flow cytometry

To determine the cell cycle status after treatment, we stained cells (10^6^ cells/condition) with 50 μg of propidium iodide and subsequently analyzed the DNA content by fluorescence-activated cell sorting (FACS). All cell cycle results were analyzed using the ModFit Software provided with the FACSCalibur flow cytometer. The relative cell cycle distribution was calculated using the FIT option. The same process was repeated for all samples.

To determine cell death, we used the Vybrant Apoptosis Assay Kit #2 (Invitrogen) (10^5^ cells/condition). The cell cycle distribution and the apoptosis assay were analyzed using ELITE Flow Cytometry (Beckman Coulter) and Cell Quest software. Duplicate assays were performed in all cases.

### Animal studies

The antiproliferative action of ascorbic acid was investigated in the HT29 xenograft mouse model. Mouse care followed the animal welfare guidelines of our institution, and local animal ethics committee approval was obtained prior to starting the experiments. Four-week-old female BalbC nude mice (Charles River, Lyon, France) (*n* = 7 per group) were subcutaneously inoculated with 1×10^6^ HT29 cells on the right flank. Ten days after implant, and once tumors had reached sizes that could be measured accurately, mice were treated with AA at the following doses: 15 mg/kg, 100 mg/kg, or 1,000 mg/kg. Drugs were administered intraperitoneally on a daily basis for 1 month. Tumor size was measured every 2 days in three dimensions using Vernier calipers, and tumor weight (g) was calculated using an ellipsoid shape to approximate the tumor mass: *m* = π/6×length×width×height. Studies were terminated after 30 days of treatment. Surviving mice have been sacrificed, and tumor weight evaluated. In addition, the date and number of surviving animals were recorded for surviving analysis.

### Statistical analysis

We used Prism v5 software to perform the statistical analysis. For tumor weight analysis we used the Mann-Whitney two-tailed statistical significance test, with a confidence interval of 95%. For the survival study the Kaplan-Meier method and log-rank tests were used. We considered a *P* value lower than 0.05 as significant.

## Results

### AA and gene expression

**Figure 1:**
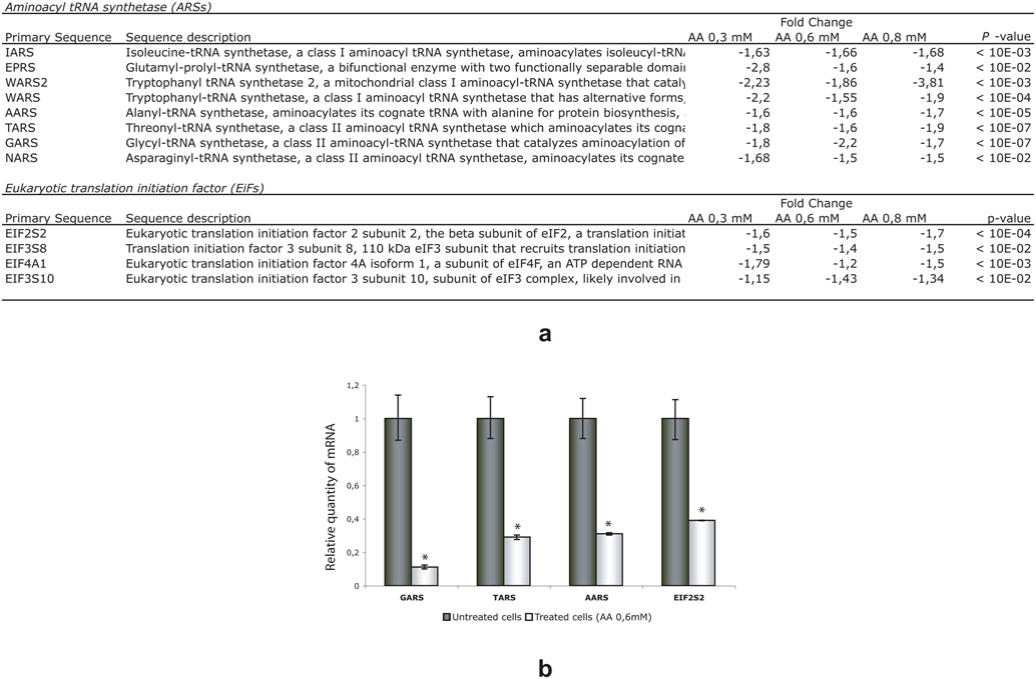
Human pangenomic microarrays and qPCR analysis. (a) Primary cultures of human skin fibroblasts were incubated in medium containing 0, 0.3, 0.6, and 0.8 mM AA. After 24 h, RNA was extracted; reverse transcribed, and hybridized on AGILENT human pangenomic microarrays. Dye swap experiments were also conducted. Data were analyzed using the Rosetta Luminator software package. And only those genes that were up- or downregulated in AA-treated cells were analyzed further. Among the downregulated genes, 40% belong to two classes (tRNA synthetases and translation initiation factor subunits) involved in protein synthesis. (b) Validation of microarray results using qPCR and Roche UPL-specific probes.

Primary cultures of normal human fibroblasts were treated for 24 h with three different concentrations of AA, ranging from 0.3 mM to 0.8 mM. RNAs were then extracted, reverse transcribed, and hybridized on AGILENT human pangenomic microarrays. RNA samples from untreated cells were labelled with Cy3, and those from treated cells were labelled with Cy5. In a reciprocal experiment, the dye labels were switched, so that untreated cells were labelled with Cy5 and treated cells with Cy3. Genes that were over- or under expressed in the initial screen and behaved in a reciprocal manner in the second screen (e.g., under expressed in the first screen and over expressed in the second screen) were selected and included in a minilibrary. Data were analyzed using Rosetta bioinformatics software. We considered only genes that varied in the same direction (up or down-expression) on all three chips with a *P* value of <0.05. Using these stringent criteria, we found that only a few genes were over expressed in cells incubated with AA, without an obvious biological function. In contrast, 31 genes appeared to be down regulated (Table S1). Of these 31 genes, 12 code for members of two protein families: tRNA synthetases and translation initiation factor subunits (Fig. 1a). These data were confirmed using qPCR (Fig. 1b).

### AA and *in vitro* cell division

**Figure 2:**
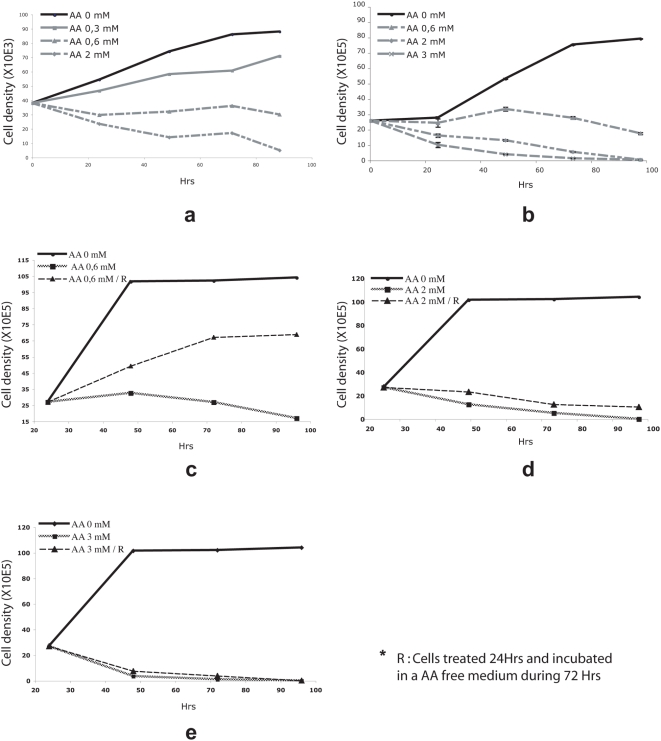
Impact of AA treatment on cell growth. Growth curves of healthy human fibroblasts (a) or Raji cells (b) incubated with various AA concentrations. Cell density estimation of the reversal of AA treatment on Raji cells incubated with 0.6 mM (c), 2 mM (d), or 3 mM (e) AA.

Both tRNA synthetases and translation initiation factor subunits are involved in protein synthesis and cell cycle progression in prokaryotes, basic eukaryotes, and mammals [6]-[11]. To determine whether AA had an impact on cell proliferation, we treated the same type of fibroblasts used in microarray assays with increasing concentrations of the drug and monitored the growth curves (Fig. 2a). At moderate concentration (0,3 mM), AA partially inhibited cell proliferation. Higher concentrations (0.6 and 2mM) respectively resulted in a cell proliferation arrest or cellular death. We then tested the effect of AA on cell lines derived from human cancers. Cell growth was affected by AA treatment in all of the cell lines, although sensitivity varied (Fig. 2b, Tables S2-S3). This difference is not understood at present. It may be related, at least in part, to varying AA uptake between cell lines since Raji cells, which are the most sensitive cells to AA treatment, also express the highest level of sodium-dependent vitamin C transporter 2 (SVCT2) mRNA (data not shown). In addition to the experiments above, we confirmed the effect of AA on cell division using a BrdU incorporation test (data not shown).

To evaluate the potential reversibility of the effect, cells incubated with increasing AA concentrations were either collected 24 h later, washed and cultured in AA-free medium, or let in presence of the drug for the next three days. We observed that the partial inhibition of cell division obtained with 0.6 mM of AA could be reversed (Fig. 2c). However, with AA doses enable to induce cell death (2 mM and 3 mM), cells continued to die even after the AA-containing medium was removed (Fig. 2d,e).

**Figure 3:**
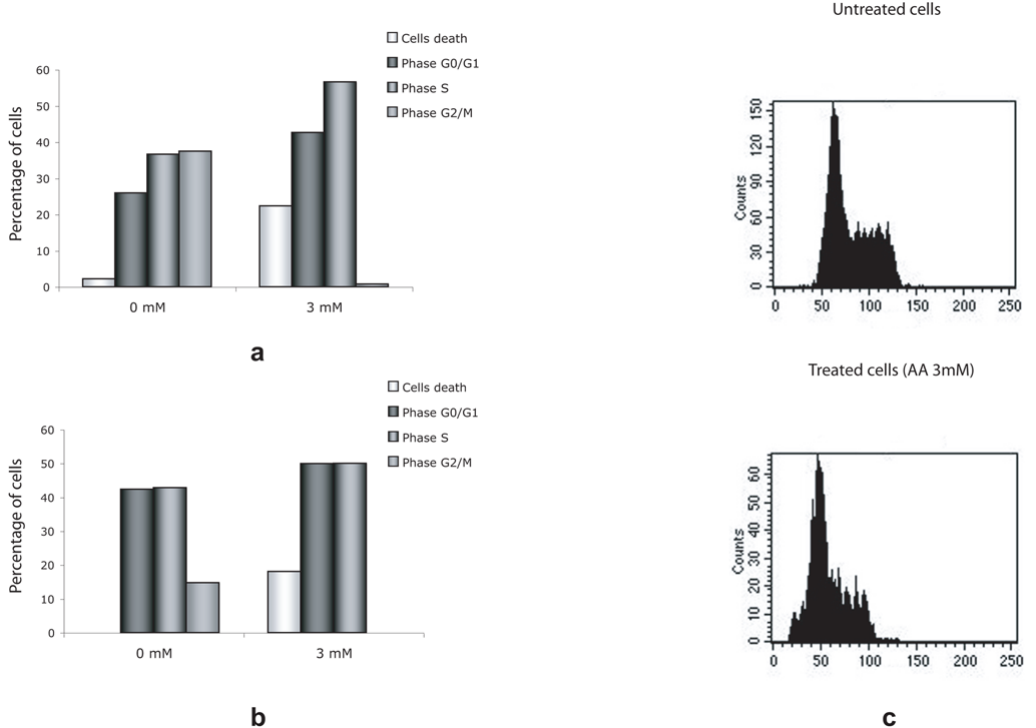
Cell cycle analysis. Cell cycles in control and treated human primary fibroblasts (a) or Raji cells (b), were analyzed using propidium iodide. DNA content was analyzed using FACS to determine the cell cycle distribution. All cell cycle results were analyzed using the ModFit software provided with the FACSCalibur flow cytometer. The FACS profile is shown in (c).

**Figure 4:**
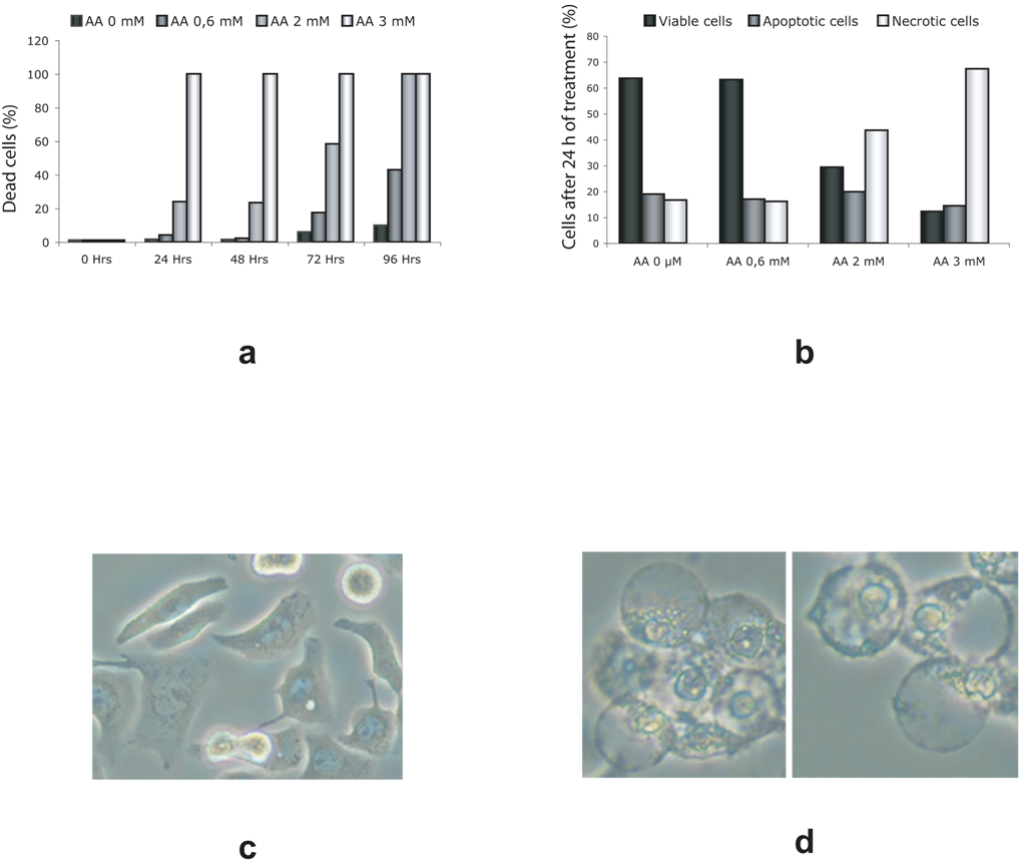
Cell viability analysis. (a) Cell viability of Raji cells treated with varying concentrations of AA was evaluated using trypan blue staining. (b) The same cells were analyzed using a propidium iodide (PI) /Annexin-V double label (see Methods). ELITE Flow Cytometry (Beckman Coulter) and Cell Quest software was used to analyze the different cell populations. Morphology of untreated (c) and treated (3mM AA) (d) colon adenocarcinoma (HT29) cells. Cells were observed with a light microscope using the phase contrast setting.

Cell cycle profiles were analyzed by FACS to determine at which stage of cell division the cells treated with AA were blocked. We noted that 3 mM AA caused a growth arrest during S phase in healthy fibroblasts cells (Fig. 3a) as well as in Raji cells (Fig. 3b, c), with no cells detected in G2/M. In addition, we observed a significant increase in cell death, which we confirmed by trepan blue uptake (Fig. 4a). To determine the type of cell death induced by AA treatment, we used flow cytometry on Raji cells, either double labelling with Annexin V (to identify the early apoptotic phase characterized by the external membrane phosphatidylserine translocation) and propidium iodide labelling (to identify plasma membrane permeabilisation related to cell necrosis) (Fig. 4b) or using a TUNEL enzymatic labelling assay of apoptosis (Fig. S1a). Nearly all of the cells treated with increasing concentrations of AA became necrotic, without an increase in apoptosis. This was confirmed by the typical swelling and bubbling morphology of the necrotic cells (Fig. 4c, d). When we treated quiescent cells (human primary fibroblasts at confluence) with the same concentration of AA, there was no effect (Fig. S1b). This data indicates that only actively proliferating cells are affected by AA treatment, and that cells are not dying from toxicity induced by high AA concentrations.

### AA and tumor progression

**Figure 5:**
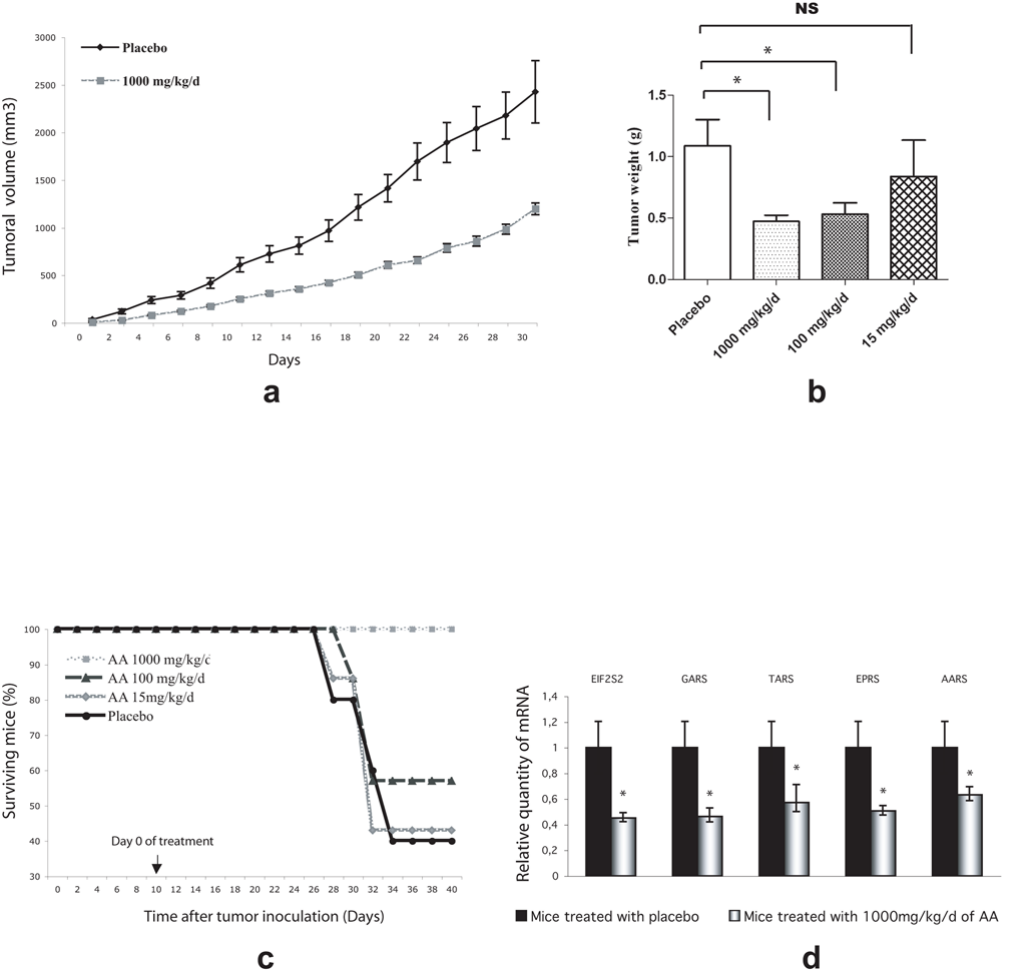
Tumor growth and weight evaluation. Kaplan Meier survival curves. Gene expression in tumor. HT29 cells were injected into nude male mice. Mice presenting with a tumor after 10 days were treated every day with either a placebo (physiological serum) or one of three AA concentrations. (a) Tumor growth in placebo-treated mice and mice treated with a daily intraperitoneal injection of AA (1000 mg/kg/d). (b) Mice were sacrificed after 1 month of a daily AA treatment and tumors were weighted to determine the effect of AA treatment. An asterisk indicates a *P* value<0,05. (c) Kaplan-Meier survival curves of mice treated with placebo or with 15 mg/kg/d, 100 mg/kg/d, or 1000 mg/kg/d AA. Once visible tumors were established (10 days after injection) mice were randomized and treated either with ascorbic acid or with placebo. Comparison of placebo treated curve and 1000 mg/kg/d treated animals, using a long rank statistical test, indicates a high statistical significance (*P* value = 0.0022). (d) Relative quantification of mRNA coding for tRNA synthetase and a translation initiation factor subunit using qPCR technology and Universal Probe Library (Roche). An asterisk indicates a *P* value<0,05.

To test the potential effect of AA treatment *in vivo*, human tumor cells were implanted by subcutaneous injection in nude mice. Colon adenocarcinoma cells (HT29) have been used since they were sensitive to AA treatment *in vitro* (Fig. S1c). Four groups of 7 grafted mice presenting visible tumors, 10 days post-implantation, were randomly separated. Animals were given a daily intraperitoneal injection during 1 month, containing either a placebo solution or AA in concentrations ranging from 15 mg/kg/d to 1000 mg/kg/d. Tumor size was evaluated every two days for 1 month (Fig. 5a). After 1 month, surviving mice were sacrificed and tumor weight was evaluated (Fig. 5b and Table S4).

Tumor growth was clearly reduced in animals receiving the highest concentration of AA (1000 mg/kg/d) when compared to the placebo group (Fig. 5a, b). All of the seven grafted mice in the 1000 mg/kg/d group were alive at the end of 1 month (Fig. 5c). On the contrary, tumor growth was not affected in the 15 mg/kg/d group (Fig. 5 b). Moreover, only three of the seven grafted mice were still alive at the end of the assay the others having died abruptly during the experiment (Fig. 5c). An intermediate situation was observed in animal treated with 100 mg/kg/d (Fig. 5b and c). A summary is presented in Table S4. The exact cause of death in animal cohorts treated either with the lower AA concentration or placebo is not easy to define. However, autopsies of animals sacrificed after 30 days or from spontaneous dead animals, revealed a massive metastatic spread with peritoneal (Fig. S2a and S2b) as well as peri hepatic carcinosis (Fig. S2c). Therefore, we propose that spontaneous death of animals that have not been treated with the highest dose of AA is due to a highly invasive carcinogenic process that is not observed in animals treated with 1000 mg/days. This suggests that AA could also protect from metastatic invasion, although this hypothesis should be confirmed in further experiments.

At the end of the treatment period, total RNAs were extracted after tumor dissection, and subjected to RT-qPCR to evaluate the deregulation of the gene expression. Consistently with the data obtained *in vitro*, tRNA synthetase and translation initiation factor subunits genes were under expressed in grafted tumors after AA treatment, in a dose-dependent manner (Fig. 5d). Hence we demonstrated that AA inhibits the expression of the same genes *in vitro* as well as *in vivo*.

## Discussion

Ascorbic acid is most well known as Vitamin C, the nutritional supplement essential for preventing scurvy. The recommended daily dose has varied over time, but it is presently about 75 to 90 mg/day. However, several authors, Linus Pauling among them, have suggested that higher daily doses might prevent cancer [12]. This has been a hotly debated topic in the scientific community. Although several papers have described the effect of AA on cell proliferation [13]-[15], the antiproliferative effects were always ascribed to the antioxidant properties of AA [13].

Based on the experiments described in this paper, we propose that AA inhibits cell division and further promotes necrosis by down-modulating the expression of genes necessary for S-phase progression. We found that actively proliferating but not quiescent cells are susceptible to AA treatment, excluding an non-specific toxicity of the highest AA concentrations. It is tempting to speculate the inhibited expression of tRNA synthetases and translation initiation elongation factor subunits leads to the rapid cessation of energy production in proliferating cells, resulting in necrotic cell death.

We have recently demonstrated that ascorbic acid is a competitive inhibitor of adenylate cyclase activity [4], resulting in a decrease of intracellular cAMP concentration. However, the mechanism underlying the regulation of tRNA synthetase and ieF subunits expression in mammalian cells are still unknown. In particular, the role of cAMP remains to be clarified.

Few experiments using animal models, with spontaneous tumors, have been performed. These studies, using an oral AA administration, report a decreased mortality for treated animals [16]. In humans, clinical trials results are mixed; some studies indicate a benefit to patients treated with AA [17], while others do not reveal any beneficial effect following AA treatment [18], [19]. We may note that positive trials involved IV injection, in contrast with negative results that involved oral administration. From data presented in this manuscript, it is obvious that treatment with increasing doses of AA induces a specific down regulation of expression of a selected set of genes, resulting in an arrest of cell proliferation and, at higher doses, in cell death.

Treatment of xenografted animals, either with a placebo or with increasing doses of AA, allowed us to draw some conclusions:

- Treatment with high doses of AA results in a lowering of tumor progression in terms of tumor weight.
- Xenografted mice treated with the highest AA concentration survive after 40 days (30 days of treatment plus 10 days of grafting). We have no explanation but we observed numerous carcinogenic invasion, in placebo treated animals and in animals treated with lower doses of AA. In contrast, animals treated with 1000 mg/days did not present carcinogenic invasion. It has been described in the literature [20]-[22] that HT29 cells are able to form metastatic carcinogenic colonies (in colon and liver). In addition, Tremblay et al demonstrate that HT29 are able to perform diapedesis.
- Genes that have been demonstrated to be down regulated in cellular in vitro experiments have also been found to be down regulated in vivo, and more precisely in tumors.

In conclusion, in terms of anticancer therapy, it appears that AA treatment will only be effective if a high enough concentration of AA can be reached (probably higher than 1mM). According to published data from healthy volunteers [23], high concentrations may only be reached by intravenous injection. These high doses have been found not to be toxic in animal as well as in human [24], [25]. Clinical trials in which patients, with advanced cancer, receiving injections of high AA doses would shed some light on the therapeutic utility of AA.

## Supporting Information

Figure S1 Tunnel enzymatic labelling assay and cell density analysis in cellular junction conditions. (a) Detection and quantification of apoptotic cells by a TUNEL assay using the In-Situ Cell Death Detection Kit, TMR red (Roche Applied Science) in accordance with the manufacturer's instructions. Analyses of apoptosis following treatment with two different AA concentrations (0.6 mM and 2 mM) were conducted on human primary fibroblasts. (b) Analysis the effect of increasing amounts of AA on confluent cultures of human primary fibroblasts. (c) Growth curves of HT29 cells (evaluated using a Neubauer hemocytometer) incubated with either 3 mM of AA or medium containing no AA.
(0.85 MB EPS)

Figure S2 Animals treated with a placebo, have been sacrificed after 30 days of treatment (plus 10 days of grafting) and autopsied. All animals present either peritoneal carcinogenic invasion (a and b) and/or peri hepatic carcinogenesis (c). Animals treated with the highest dose did not present any invasion.
(4.47 MB EPS)

Table S1 (0.05 MB DOC)

Table S2 (0.02 MB DOC)

Table S3 (0.02 MB DOC)

Table S4 (0.02 MB DOC)
