# Supplementary material for: Antiproliferative Effect of Ascorbic Acid Is Associated with the Inhibition of Genes Necessary to Cell Cycle Progression
Source: PLoS One. 2009 Feb 6;4(2):e4409. doi: 10.1371/journal.pone.0004409 (PMC2634969; doi:10.1371/journal.pone.0004409)
Supplement: Table S1 — (0.05 MB DOC) [file pone.0004409.s003.doc]

# Table S1. Genes underexpressed after AA treatment

| ***Aminoacyl tRNA synthetase (ARSs)*** | | |  |  |
| --- | --- | --- | --- | --- |
|  | Fold Change | | |  |
| Primary Sequence | **AA 0,3 mM** | **AA 0,6 mM** | **AA 0,8 mM** | ***P*-value** |
| IARS | -1,63 | -1,66 | -1,68 | < 10E-03 |
| EPRS | -2,8 | -1,6 | -1,4 | < 10E-02 |
| WARS2 | -2,23 | -1,86 | -3,81 | < 10E-03 |
| WARS | -2,2 | -1,55 | -1,9 | < 10E-04 |
| AARS | -1,6 | -1,6 | -1,7 | < 10E-05 |
| TARS | -1,8 | -1,6 | -1,9 | < 10E-07 |
| GARS | -1,8 | -2,2 | -1,7 | < 10E-07 |
| NARS | -1,68 | -1,5 | -1,5 | < 10E-02 |
|  |  |  |  |  |
| ***Eukaryotic translation initiation factor (EiFs)*** | | | |  |
|  | **Fold Change** | | |  |
| **Primary Sequence** | **AA 0,3 mM** | **AA 0,6 mM** | **AA 0,8 mM** | ***P*-value** |
| EIF2S2 | -1,6 | -1,5 | -1,7 | < 10E-04 |
| EIF3S8 | -1,5 | -1,4 | -1,5 | < 10E-02 |
| EIF4A1 | -1,8 | -1,2 | -1,5 | < 10E-03 |
| EIF3S10 | -1,15 | -1,43 | -1,34 | < 10E-02 |
|  |  |  |  |  |
| Apoptosis and Cellular Stress | | | |  |
|  | **Fold Change** | | |  |
| **Primary Sequence** | **AA 0,3 mM** | **AA 0,6 mM** | **AA 0,8 mM** | ***P*-value** |
| HSPA5 | -1,8 | -1,6 | -1,8 | < 10E-05 |
| HSPA9B | -1,6 | -1,4 | -1,7 | < 10E-03 |
| HERPUD1 | -1,7 | -1,8 | -1,8 | < 10E-05 |
| C20orf97 | -5,8 | -6,5 | -5,3 | < 10E-05 |
| DKK1 | -1,7 | -1,5 | -1,6 | < 10E-04 |
|  |  |  |  |  |
| Miscellanous |  |  |  |  |
|  | **Fold Change** | | |  |
| **Primary Sequence** | **AA 0,3 mM** | **AA 0,6 mM** | **AA 0,8 mM** | ***P*-value** |
| ASNS | -4,1 | -4,4 | -4,3 | < 10E-15 |
| LOXL2 | -1,5 | -1,5 | -1,4 | < 10E-05 |
| SERPINB2 | -2,4 | -1,5 | -2,5 | < 10E-02 |
| PSA | -3,2 | -3,8 | -3,2 | < 10E-15 |
| SHMT2 | -2,2 | -2,1 | -2 | < 10E-06 |
| SMC6L1 | -1,7 | -1,5 | -1,7 | < 10E-06 |
| PDIP | -4,1 | -3,9 | -7,9 | < 10E-04 |
| PHGDH | -2,2 | -2,6 | -2,1 | < 10E-12 |
| CBS | -1,9 | -2,1 | -1,8 | < 10E-05 |
| P4HA2 | -1,7 | -1,7 | -1,7 | < 10E-04 |
| MTHFD2 | -3,1 | -3,8 | -3,2 | < 10E-05 |
| DKK1 | -1,7 | -1,6 | -1,5 | < 10E-05 |
| ARHE | -1,5 | -1,3 | -1,5 | < 10E-05 |
| PCK2 | -3,2 | -3 | -3,2 | < 10E-07 |
