# Supplementary material for: Antiproliferative Effect of Ascorbic Acid Is Associated with the Inhibition of Genes Necessary to Cell Cycle Progression
Source: PLoS One. 2009 Feb 6;4(2):e4409. doi: 10.1371/journal.pone.0004409 (PMC2634969; doi:10.1371/journal.pone.0004409)
Supplement: Table S2 — (0.02 MB DOC) [file pone.0004409.s004.doc]

**Table S2. Effect of a high ascorbic acid concentrationon cell proliferation.**

|  | **Primary fibroblasts (GM00038)** | | **Colon Adenocarcinoma cells (HT29)** | | **Plasmocytoma cells (CCL155)** | | **Lymphoma cells (Raji)** | |
| --- | --- | --- | --- | --- | --- | --- | --- | --- |
|  | Placebo | **3 mM AA** | **Placebo** | **3 mM AA** | **Placebo** | **3 mM AA** | **Placebo** | **3 mM AA** |
| Cell number (10^4^) ***t* = 0** | 25 | 25 | 25 | 25 | 250 | 250 | 250 | 250 |
| Cell number  (10^4^) ***t* = 72** | 56 | 11 | 212 | 70 | 650 | 170 | 600 | 5 |

Normal cells (healthy human primary skin fibroblasts) or neoplastic cell lines (HT29 from human colon adenocarcinoma, Raji from Burkitt’s lymphoma, or CCL155 from plasmocytoma) were incubated either with placebo or with 3 mM of AA for 72 h. Cells were counted at the beginning of the incubation (*t* = 0) and after 72 h (*t* = 72).
