# Supplementary material for: Antiproliferative Effect of Ascorbic Acid Is Associated with the Inhibition of Genes Necessary to Cell Cycle Progression
Source: PLoS One. 2009 Feb 6;4(2):e4409. doi: 10.1371/journal.pone.0004409 (PMC2634969; doi:10.1371/journal.pone.0004409)
Supplement: Table S3 — (0.02 MB DOC) [file pone.0004409.s005.doc]

**Table S3. Impact of increasing concentration of AA on Raji cells proliferation. Cell number x 10^5^**

| **Hrs** | AA 0 µM | **AA 0,6 mM** | **AA 2 mM** | **AA 3 mM** |
| --- | --- | --- | --- | --- |
| 0 | 25+/- 0 | 25+/- 0 | 25+/- 0 | 25+/- 0 |
| 24 | 29,35+/-2,14 | 23,62+/-3,84 | 16+/-0,89 | 8,32+/-3,13 |
| 48 | 50,97+/-0,54 | 35,99+/-2,04 | 18,23+/-0,62 | 2,45+/-0,34 |
| 72 | 60,33+/-2,23 | 25,24+/-3,80 | 9,58+/-0,69 | 0,47+/-0,12 |
| 96 | 62,62+/-0,26 | 15,87+/-1,90 | 1,18+/-1,94 | 0+/-0 |

Raji cells cells have been incubated without or with increasing AA concentration, in a classical incubation medium. Cells have been counted in triplicate each 24 hrs during 96 hours.
