# Supplementary material for: Antiproliferative Effect of Ascorbic Acid Is Associated with the Inhibition of Genes Necessary to Cell Cycle Progression
Source: PLoS One. 2009 Feb 6;4(2):e4409. doi: 10.1371/journal.pone.0004409 (PMC2634969; doi:10.1371/journal.pone.0004409)
Supplement: Table S4 — (0.02 MB DOC) [file pone.0004409.s006.doc]

**Table S4. Tumor weight and number of surviving mice after 30 days of AA treatment.**

| Treatment | Tumor weight after 30 days (g) | *P* value | Number of surviving mice after 30 days |
| --- | --- | --- | --- |
| Placebo | 1.089 +/− 0.2138 | — | 3/7 |
| 1,000 mg/kg/d | 0.473 +/− 0.050 | 0.0111 | 7/7 |
| 100 mg/kg/d | 0.531 +/− 0.092 | 0.0452 | 4/7 |
| 15 mg/kg/d | 0.838 +/− 0.295 | 0.5312 | 3/7 |

Nude mouse have been grafted with HT29 cells and maintained in P2 sterile conditions. Tumors from animals treated with either a placebo or with increasing concentrations of AA were excised and weighed. Means and standard error were calculated. The statistical significance in tumor weight of AA-treated versus placebo-treated tumors was evaluated using the Mann-Whitney two-tailed test (95% significance). The number of surviving mice following 30 days of treatment is indicated. Survival analysis was done using Kaplan-Meier method and log-rank tests, see Fig 5c.
